# Introducing untargeted data-independent acquisition for metaproteomics of complex microbial samples

**DOI:** 10.1038/s43705-022-00137-0

**Published:** 2022-06-29

**Authors:** Sami Pietilä, Tomi Suomi, Laura L. Elo

**Affiliations:** 1 Turku Bioscience Centre, University of Turku and Åbo Akademi University, FI-20520 Turku, Finland; 2 Institute of Biomedicine, University of Turku, FI-20520 Turku, Finland

**Keywords:** Proteomics, Microbial communities

## Abstract

Mass spectrometry-based metaproteomics is a relatively new field of research that enables the characterization of the functionality of microbiota. Recently, we demonstrated the applicability of data-independent acquisition (DIA) mass spectrometry to the analysis of complex metaproteomic samples. This allowed us to circumvent many of the drawbacks of the previously used data-dependent acquisition (DDA) mass spectrometry, mainly the limited reproducibility when analyzing samples with complex microbial composition. However, the DDA-assisted DIA approach still required additional DDA data on the samples to assist the analysis. Here, we introduce, for the first time, an untargeted DIA metaproteomics tool that does not require any DDA data, but instead generates a pseudospectral library directly from the DIA data. This reduces the amount of required mass spectrometry data to a single DIA run per sample. The new DIA-only metaproteomics approach is implemented as a new open-source software package named *glaDIAtor*, including a modern web-based graphical user interface to facilitate wide use of the tool by the community.

## Introduction

Microbiome profiling has attained increasing attention in the past few years with the recognition of the important role of microbiota in human health and disease [1–3] and potential major implications for disease prediction, prevention, and treatment. Currently, metagenome sequencing has remained the most common approach to study microbiome, with several successful applications in various studies, including large multi-center studies of thousands of samples using either 16S rRNA or whole genome sequencing [4]. By cataloguing which microbes are present in a sample and their relative abundances, metagenomics can provide important information about the taxonomic composition of the microbial communities and predict their functional potential. A major limitation of the metagenome approach is, however, that it does not directly assess the function of the microbiota. To overcome this limitation, mass spectrometry based metaproteome analysis has emerged as a compelling option.

Metaproteomics is a relatively new field of research that aims to characterize all proteins expressed by a community of microorganisms in a complex biological sample [5]. Its major promise lies in its ability to directly measure the functionality of microbiota, while the more widely used metagenomics captures only the taxonomic composition and functional potential. Therefore, metaproteomics has emerged as an intriguing option, for example, in the study of human gut microbiota functionality in various healthy and disease states [6, 7].

To date, metaproteomics has typically involved data-dependent acquisition (DDA) mass spectrometry, which is, however, known to have limitations [8]. For example, only the most intense peptide ions are selected for fragmentation, which leaves the rest of the peptides unidentified, while MS1-based methods still allow their quantification. This is particularly challenging for metaproteomics, where the vast number of peptides increase the chance of co-elution. The selection also introduces stochasticity to the identifications, reducing the overlap between repeated analyses. For this reason, DDA often requires multiple runs from the same sample to discover all obtainable peptides. Furthermore, the ion intensities are often not consistently recorded through the whole chromatographic profile, making quantification challenging [9].

To overcome the limitations of DDA, data-independent acquisition (DIA) mass spectrometry systematically fragments all precursor peptide ions. Therefore, DIA has been proposed as an alternative method to overcome many fallbacks of DDA. However, the systematic fragmentation of the precursor peptide ions produces highly convoluted fragment spectra, making peptide identification a difficult task. This is especially pronounced for complex metaproteomic samples, where multiple precursor ions are more likely to elute simultaneously.

Recently, we were the first to demonstrate that DIA mass spectrometry can be successfully applied to analyze complex metaproteomic samples by using a spectral library constructed from corresponding DDA data to assist the peptide identification [10]. While such DDA-assisted DIA method requires the peptides to be previously discovered through DDA, it allows reproducible identification and quantification of the detected peptides across the samples [11]. However, the requirement for having a DDA-based spectral library can be considered as a major drawback of the method. Creating the DDA-based spectral library consumes sample material, may not represent well the content of all the samples and, most importantly, brings the DDA-related limitations of peptide identification to DIA, as only peptides present in the library can be detected from the DIA data.

To this end, we introduce here untargeted analysis of DIA metaproteomics data without the need for any DDA data. To solve the problem of convoluted DIA spectra, we generate a pseudospectral library directly from the DIA data. This is done using the DIA-Umpire algorithm to deconvolve the DIA spectra into DDA-like pseudospectra, having precursors and their fragments, which can then be used for peptide identification with conventional protein database searches [12]. Similar approach has not been used in complex metaproteomics studies before. Using a laboratory-assembled microbial mixture and human fecal samples, we demonstrate that our DIA-only metaproteomic approach enables overcoming the limitations of the DDA-assisted DIA approach and reduces the number of required mass spectrometry analyses to a single DIA analysis per sample.

The new DIA-only metaproteomics approach is implemented as a new open-source software package named *glaDIAtor*. It contains two different interfaces to facilitate wide use of the tool by the community. The easy-to-use graphical user interface (GUI) is suited to users without extensive bioinformatics background, whereas the command line interface is more suited to high-performance computing (HPC) cluster usage and other scripted use cases. To provide a modern graphical web user interface, *glaDIAtor* utilizes the Pyramid and Vue frameworks. The primary intended method for its deployment is a server installation, where it can be accessed from multiple workstations by using web browsers, such as Firefox or Chrome. Alternatively, *glaDIAtor* can be deployed to a workstation where the web service is visible only to the local machine. Using the command line interface, *glaDIAtor* can be deployed on HPC clusters under work managers such as SLURM.

## Results

**Fig. 1:**
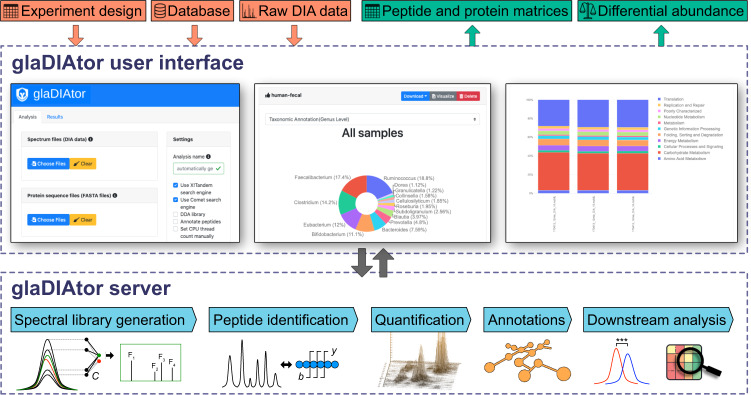
Schematic illustration of the glaDIAtor workflow and software. *glaDIAtor* is an open-source software package that implements a complete workflow for DIA metaproteomics data from raw mass spectrometry files to peptide quantifications and their taxonomic and functional annotations (lower panel). To enable wide use of the tool, we provide both a modern web-based graphical user interface (upper panel) as well as a command line interface that enables high-performance computing (HPC) cluster usage.

The *glaDIAtor* software package implements a complete data analysis workflow for DIA metaproteomics from raw mass spectrometry files to peptide quantification and taxonomic/ functional annotation (Fig. 1). It is implemented using container technology, which provides all the required utilities and libraries in a single package enabling easy installation on multiple different platforms [13, 14], including support for server and workstation deployments. To enable broad adoption of the tool, we provide both a modern web-based graphical user interface as well as a command line interface that enables HPC cluster usage.

To demonstrate the feasibility and benefits of our new DIA-only metaproteomics approach, we applied it to a laboratory-assembled microbial mixture containing twelve different bacterial strains (12mix) and to human fecal samples from six healthy donors (Supplementary Tables 1 and 2).

### Peptide identifications with glaDIAtor DIA metaproteomics are highly reproducible

**Fig. 2:**
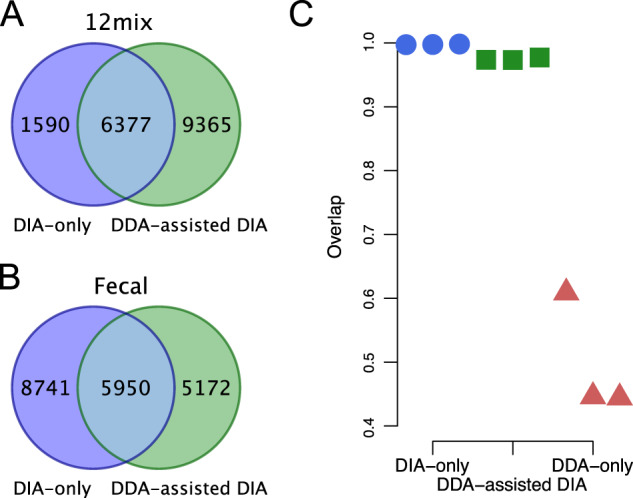
Overlap of the peptides detected by the DIA-only and the DDA-assisted DIA approach. Overlap of the **A** 12mix and **B** human fecal samples. **C** Overlap of the detected peptides between all pairs of replicated 12mix samples using the DIA-only, the DDA-assisted DIA, and the DDA-only approach.

First, we investigated the peptide identification yields using the DIA-only approach of *glaDIAtor* and compared them against our previously introduced DDA-assisted DIA method [10] and the conventional approach of using DDA only. In the simplified 12mix data, three replicate samples were prepared from the same bacterial mixture and analyzed separately with DIA. For the generation of the DDA-based spectral library, these three replicates were pooled together and analyzed in triplicate. Since both the individual replicates and the pooled sample represented the same mixture, they were highly similar. As expected, this was advantageous for the DDA-assisted DIA approach and, accordingly, it identified more peptides than the DIA-only approach in these data (15742 vs. 7967 peptides, Fig. 2A). Interestingly, however, we also identified a considerable number of peptides using the DIA-only method. On average, 7957 peptides per sample were identified using the DIA-only approach, with 7943 peptides identified across all the replicates. With the DDA-assisted DIA approach, on average, 15519 peptides were identified per sample, with 15218 peptides identified across all the replicates. Use of only the DDA data identified, on average, 18132 peptides per sample, while the number of peptides identified across all the replicates was 10912.

In the complex human fecal data, six replicate samples were prepared from different individual donors and analyzed separately with DIA. For the generation of the DDA-based spectral library, these six samples were pooled together and analyzed with six injections to increase the peptide coverage in the library. Importantly, in these complex data, the DIA-only approach produced over 30% more peptide identifications than the DDA-assisted DIA approach (14691 and 11122 peptides, Fig. 2B). On average, 8211 peptides per sample were identified using the DIA-only approach and 6385 peptides using the DDA-assisted DIA approach. Use of only the pooled DDA data identified, on average, 18650 peptides per technical replicate, while the total number of peptides identified across all the technical replicates was 7348.

Of all the peptides identified by either the DDA-assisted DIA or the DIA-only approach, 37% and 30% were shared between both approaches in the 12mix and the fecal data, respectively (Fig. 2A, B). This was in line with the observed overlap of ~35% between the corresponding spectral and pseudospectral libraries, which were used as the search space for the peptides and determined what could be identified from the DIA data (Supplementary Fig. 1). Of note, while deconvolution is able to extract pseudospectra from the DIA data, the set of fragment ions for a given peptide may not be exactly the same as with DDA, but even peptides with identical amino acid sequences can be represented by partially different sets of fragment ions between the libraries, possibly affecting the peptide identification (Supplementary Fig. 2).

Since the 12mix data contained technical replicates, it also allowed us to investigate the technical reproducibility of the identified peptides. This was done by comparing the overlap of the peptide identifications between each possible pair of replicates divided by the total number of peptides identified in the replicate pair. The reproducibility of both the DIA-only and the DDA-assisted DIA approach was high, with an overlap of over 99% and 96%, respectively (Fig. 2C), whereas the use of only the DDA data of the pooled mixture samples resulted in an overlap of only ~40% between technical repeats, highlighting the improved reproducibility of DIA over DDA.

### DIA-only approach by glaDIAtor enables detection of individual-specific microbial taxonomic profiles

**Fig. 3:**
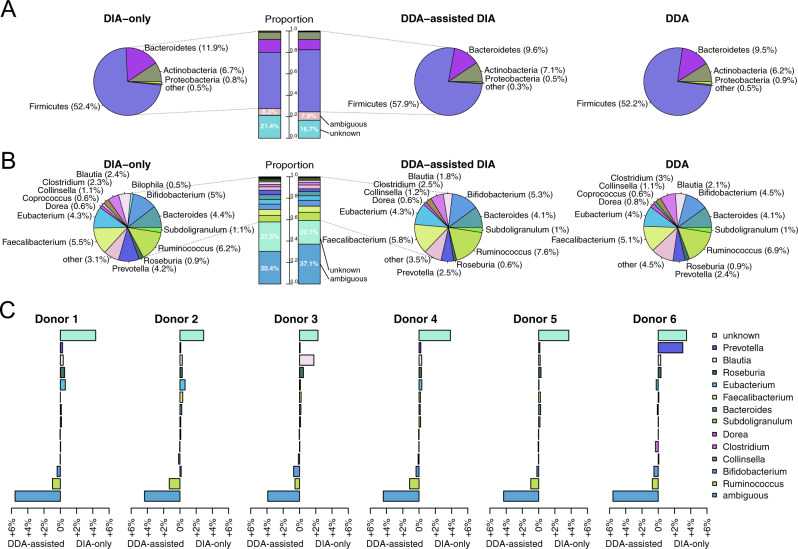
Taxonomic profiles of the human fecal samples. **A** Phylum-level and **B** genus-level taxonomic annotations of the peptides using the DIA-only approach (left panel), the DDA-assisted DIA approach (middle panel), or only the pooled DDA data (right panel). Phyla or genera having less than 0.5% of the total peptides were aggregated to category *other*. **C** Differences between the DIA-only and the DDA-assisted DIA results of the individual human fecal samples, measured by the differences of the observed percentages between the two approaches.

The overall taxonomic profiles of the metaproteomes observed were mostly similar when using the DIA-only and the DDA-assisted DIA approach, as well as only the DDA data. With all approaches, a unique taxonomic annotation was assigned at phylum level to >90% of the peptides in the 12mix and ~70% of the peptides in the human fecal data (Fig. 3A, Supplementary Fig. 3A). At genus level, a unique taxonomic annotation was assigned with all approaches to ~60% of the peptides in the 12mix and ~40% of the peptides in the human fecal data (Fig. 3B, Supplementary Fig. 3B). Only ~2% of the identified peptides in the laboratory assembled 12mix were annotated to genera not present in the mixture, while the taxonomic profile of human fecal samples was similar to those reported in the literature, [6, 15]. Similarly, despite the differences in the spectral and pseudospectral libraries, the overall taxonomic profiles of their overlapping and non-overlapping parts remained similar (Supplementary Fig. 4).

To take a closer look at the differences between the DIA-only and DDA-assisted DIA approaches, we compared the genus-level taxonomic profiles of the peptides in the human fecal data. Although the overall taxonomic profiles were highly similar, some notable differences were observed. First, while the DIA-only approach detected a larger number of peptides, it also detected a larger proportion of peptides that did not yet have taxonomic annotation in the widely used integrated reference catalog of the human gut microbiome (IGC) [16]. Secondly, the DDA-assisted DIA method detected a larger proportion of ambiguous peptides with multiple different annotations in IGC, which is typical to peptides that are shared by multiple organisms. Finally, when investigating the individual genera, the largest difference between the results of the DIA-only and DDA-assisted DIA approaches was observed in *Prevotella* in the human fecal samples, which had a 4.2% share with the DIA-only approach and 2.5% share with the DDA-assisted DIA approach. A closer look at the individual human fecal samples revealed that *Prevotella* was dominant in a single sample, while its proportion in the other samples was very low (Fig. 3C). This illustrates the possible limitations of a pooled DDA library in the presence of large individual variation in the metaproteomes.

### DIA-only approach by glaDIAtor enables detection of individual-specific microbial functional profiles

**Fig. 4:**
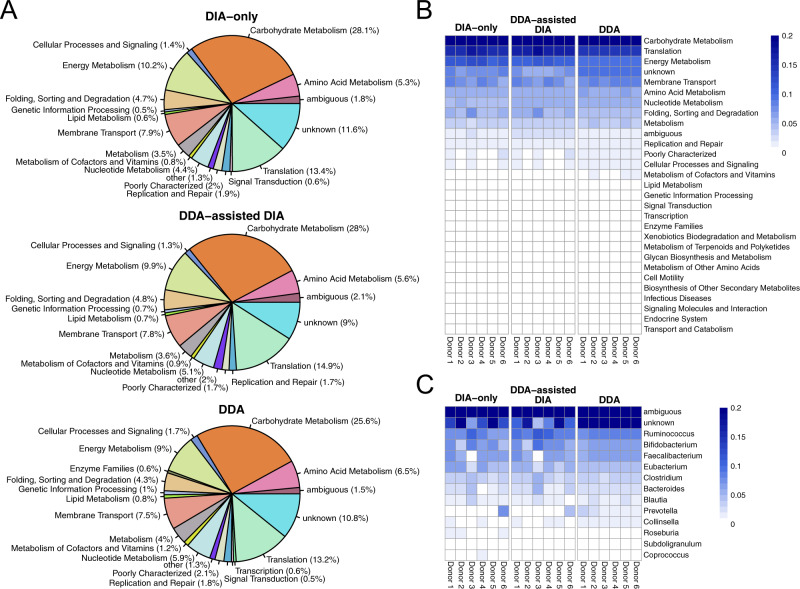
Functional profiles of the human fecal samples. **A** KEGG functional categories of the peptides using the DIA-only approach (top panel), the DDA-assisted DIA approach (middle panel), or only the pooled DDA data (bottom panel). Functional categories having less than 0.5% of the total peptides were aggregated to category *other*. **B** Heatmaps of the functional profiles of the six individual healthy donor samples using the DIA-only and the DDA-assisted DIA approach, as well as the six technical repeats of the pooled sample analyzed with DDA. **C** Genus-wise distributions of the peptides involved in carbohydrate metabolism in the six individual healthy donor samples using the DIA-only and the DDA-assisted DIA approach, and in the six technical repeats of the pooled sample analyzed with DDA.

Next, we investigated the functional profiles of the metaproteomes observed using the DIA-only, the DDA-assisted DIA, or only the DDA data. Using KEGG functional categories from the IGC database, a functional annotation could be assigned to ~90% of the peptides in both the 12mix and the human fecal data with all the approaches (Fig. 4A, Supplementary Fig. 5A), supporting the utility of the peptide-centric approach in the functional annotations without the need for the intermediate step of protein inference. The overall functional profiles with all the approaches were highly similar (Fig. 4A, Supplementary Fig. 5A, B). Similarly, despite the differences in the spectral and pseudospectral libraries, the overall functional profiles of their overlapping and non-overlapping parts remained similar (Supplementary Fig. 6).

A more detailed examination of the functional profiles in the human fecal data suggested that the largest functional categories included carbohydrate metabolism (~30%), translation (~15%), and energy metabolism (~10%), which together covered over 50% of the peptides (Fig. 4A). These are in line with previous studies of healthy human fecal metaproteomes [17, 18]. The overall functional profiles were highly similar across the different individuals and with all the different approaches, despite differences in the peptide identifications (Fig. 4B). This was also true to the detected functional pathways, despite differences in the coverage of the pathways (Supplementary Fig. 7). However, more detailed investigation of the functionality can reveal more variation between the individuals. For instance, by investigating genus-wise distribution of the peptides involved in carbohydrate metabolism, the individual with the largest *Prevotella* abundance stood out, as well as did another individual with markedly lower contribution of *Faecalibacterium* compared to the other individuals (Fig. 4C).

### DIA metaproteomics using glaDIAtor enables reproducible peptide quantifications

**Fig. 5:**
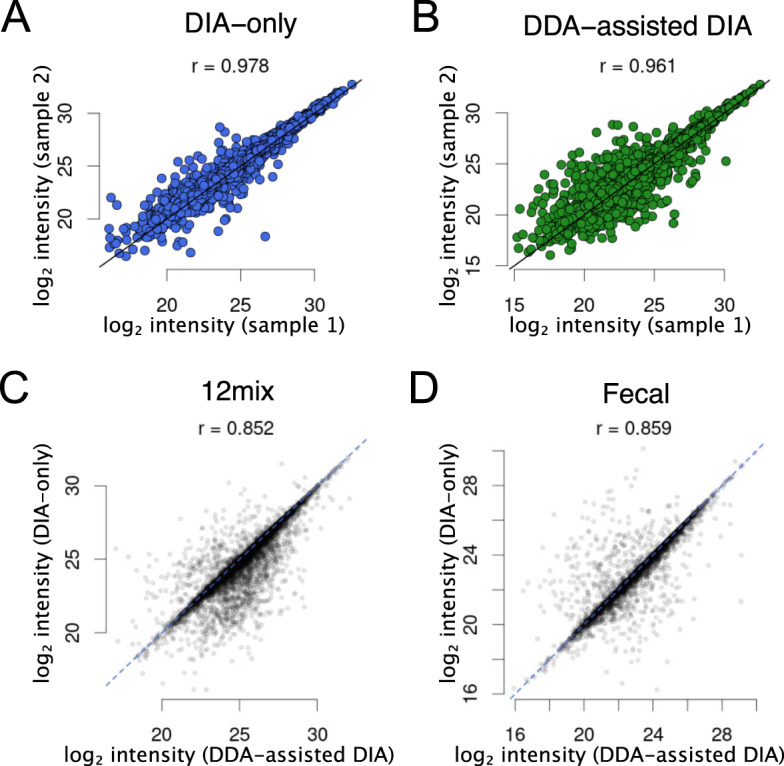
Representative examples of correlations of peptide quantifications. Correlations between two technical replicates of the 12mix samples using **A** the DIA-only or **B** the DDA-assisted DIA approach, or between the DIA-only and the DDA-assisted DIA approach in the **C** 12mix and **D** human fecal samples. All the corresponding pairwise comparisons are shown in Supplementary Figs. 4 and 5.

Finally, we assessed the performance of the DIA-only approach of *glaDIAtor* in quantifying the identified peptides. The pairwise Pearson correlation coefficients between the quantifications across the technical replicates in the 12mix data were very high (*r* > 0.97 with *p* < 0.001 in each pairwise comparison, Fig. 5A, Supplementary Fig. 8), indicating high reproducibility. The values were even higher than with the DDA-assisted DIA approach, which already produced highly reproducible quantifications (*r* > 0.95 with *p* < 0.001 in each pairwise comparison, Fig. 5B, Supplementary Fig. 8).

Comparison of the quantifications between the DIA-only and the DDA-assisted DIA approach across the shared peptides suggested overall high correlations in both the 12mix and the human fecal samples (*r* > 0.85 with *p* < 0.001 in each comparison) (Fig. 5C, D, Supplementary Fig. 9). The differences were related to the libraries built by the approaches, where each unique peptide can be represented by multiple ions with different charge states and modifications and the same peptide is typically represented by a partially different set of fragments. Since the peptides are quantified based on the fragment level, this results in quantification differences between the approaches.

## Discussion

A major bottleneck in the utilization of mass spectrometry metaproteomics has been the lack of appropriate computational tools to interpret the data produced [19]. Because of the inherent complexity of the data, conventional tools to analyze single-species proteome data are often not well suited for metaproteomics. While a few tools have been introduced for the analysis of DDA metaproteome samples [20, 21], until our recent work [10], there have been no tools for DIA mass spectrometry metaproteomics, despite its high potential to improve the reproducibility over the DDA mode. Here, we introduce untargeted DIA for metaproteomics of complex microbial samples, which is a major practical improvement to metaproteomics. Importantly, we demonstrate that reproducible identification and quantification of microbial peptides is possible without the need for any additional DDA library samples using our new DIA-only tool *glaDIAtor*.

In comparison to our previously introduced DDA-assisted DIA approach, a major benefit of the proposed DIA-only approach is that it omits the need to design a representative set of DDA samples for spectral library generation and, thereby, it reduces the number of samples that need to be analyzed. This is of particular importance in studies with large numbers of samples, such as those in clinical study settings.

In addition to a reduced number of samples that need to be analyzed, the new DIA-only approach also circumvents the initial DDA-originated limitations in peptide identification that hamper approaches using a DDA-based spectral library, especially as such library is typically prepared using only a limited set of pooled samples. For instance, with the complex human fecal data, the DIA-only approach improved the number of peptide identifications over the DDA-assisted DIA approach. This supports the notion that a spectral library generated from pooled DDA samples might not represent well the whole diversity of the samples but is dominated by highly abundant peptides and peptides commonly detected in the samples. This may play a crucial role in the data interpretation; for instance, in the analysis of the human fecal samples, *Prevotella* was dominant in one of the samples but lowly abundant in the others. If the DDA library does not represent well the whole sample set, important parts of the microbial communities may remain undetected, which can be avoided with the proposed DIA-only approach. Still, a comprehensive DDA library can enable detection of peptides in greater numbers than is detected by deconvolving the DIA data (Fig. 2A), suggesting that there likely remains room for further methodological improvements for deconvolution, as not all peptides present in the DIA-data are detected.

A well-known limitation of the DDA-based analysis is that it tends to detect peptides that are highly abundant. This includes peptides shared by multiple species, which causes a peptide to have an ambiguous taxonomic annotation. Accordingly, we observed a relatively large proportion of peptides with ambiguous taxonomic annotation with the DDA-assisted DIA approach at the genus level. On the other hand, the DIA-only approach tended to detect a larger proportion of peptides with unknown genus annotation than the DDA-assisted DIA approach, indicating that they were less well characterized in the current databases. This suggests that the most abundant proteins, commonly targeted by DDA, are more likely to have an annotation in the database. This may also suggest the ability to target proteins not detectable by other means, thus revealing proteins that are not well known and annotated by databases.

In the current version of *glaDIAtor*, peptide annotations are assigned to fixed taxonomy levels using a peptide-centric approach. Alternatively, the widely used lowest common ancestor (LCA) approach could be used for taxonomic annotation, which maps the peptides to the taxonomic lineages based on the lowest common ancestor [22]. The same peptide-centric approach is also applicable to functional annotations, which are typically not organized in tree-based structures, making the LCA approach unsuitable for them. A benefit of our pragmatic peptide-centric approach is that it allows examination of the data at different levels of generality, instead of only the most specific annotation(s) associated with a peptide, such as the most specific taxon with LCA.

Technically, the analysis of metaproteomics data can be a very computationally intensive task in terms of the required computing power and memory usage. For building a spectral or pseudospectral library, a great deal of computing power is needed to compare the theoretical spectra of the vast sequence database against the experimental spectra obtained from the DIA data. With the datasets in this study, the library generation was found to be the most time-consuming step that can take several days. By comparison, the DIA-only approach was computationally more intensive than the DDA-assisted DIA approach due to the fact that each DIA spectrum file was deconvoluted to pseudospectrum files, which were subsequently analyzed to build a pseudospectral library. Once the spectral or pseudospectral library has been produced, the subsequent analysis of the DIA data against the library spectra is then considerably faster. The current version of *glaDIAtor* scales the processing with threads using efficiently the processing power of a single computer. However, it is possible to extend the parallel analysis to multiple computers, such as cluster environments. On AMD EPYC hardware with 64 cores and 228GB RAM, the *glaDIAtor* run times for both the 12mix and human fecal datasets were ~12 h in DDA-assisted DIA mode and ~24 h in DIA-only mode. To enable easy deployment of *glaDIAtor*, it is implemented as a software container which provides all the required utilities and libraries in a single package.

An interesting future development would be to circumvent the need of generating reference spectra separately for each new project. For this, machine learning has been suggested as a possible solution using, for instance, artificial neural networks [23–25]. A major challenge with such approaches is, however, their potential biases towards the training data and need for re-training for specific conditions. This remains an interesting topic for further investigation. In general, the microbiome research still involves multiple different types of unknowns that are continuously being revealed thanks to improved technologies [26], with metaproteomics providing an excellent opportunity to uncover the functional aspects of the microbial communities.

## Materials and methods

### Generation of DIA-based pseudospectral library in glaDIAtor

To enable building a pseudospectral library directly from the DIA data, the DIA spectra were deconvolved into pseudospectra containing precursor ions and their corresponding fragment spectra, following a similar procedure as previously proposed for single-species proteomics [12]. In short, a two-dimensional feature detection algorithm was first used to locate precursor and fragment ions from the MS1 and MS2 data. Pearson correlation coefficients of the elution peaks and retention time differences of the peak apices were then used to group the fragment ions with the precursors. For the generation of a pseudospectrum for each precursor-fragment group, all likely complementary *y* and *b* ions were detected. Finally, the obtained DIA pseudospectra were further filtered by searching them with X!Tandem [27] and Comet [28] algorithms against the *Integrated reference catalog of the human gut microbiome* (IGC, 9.9M) [16], containing over 9 million protein sequences covering human gut bacteria. The false discovery rate (FDR) for the identifications was set at 1%. The identified spectra formed the final pseudospectral library that was used to identify peptides from the DIA data.

### Peptide identification and quantification

For peptide identification, either the DIA-based pseudospectral library (referred to as DIA-only approach) or the DDA-based spectral library (referred to as DDA-assisted DIA approach) was used. Both libraries utilized X!Tandem [27] and Comet [28] algorithms and the IGC reference database for peptide identification. Parent ion mass tolerance was set to 10 ppm and fragment ion tolerance to 0.02 Da. The false discovery rate (FDR) for the spectral library matching was set at 1%. For TRIC feature alignment [29], the target and maximum FDRs were set to 1% and 5%, respectively.

### Taxonomic and functional annotations

The identified peptides were taxonomically and functionally annotated using the annotations from the IGC database without protein inference. For each peptide, annotations of all possible protein sequences were retrieved. Each peptide was first assigned to all its possible source proteins. The annotation of a peptide was then determined on the basis of the annotations of these proteins. If all the source proteins had the same annotation, then that annotation was assigned to the peptide. Unannotated source proteins were allowed since they do not conflict with the existing annotations. In case the source proteins had different annotations, then the peptide annotation was labeled as ambiguous.

### glaDIAtor software and availability

The *glaDIAtor* software is open source and distributed as a Docker image that can be downloaded from DockerHub repository elolab/gladiator. The current image is based on Ubuntu 20.04 and comes bundled with several programs and libraries that retain their original licenses. The installed software include: Comet 2019.01 rev. 5, X!Tandem 2017.02.01.4, OpenMS 2.4 (includes OpenSWATH), Trans-Proteomic Pipeline (TPP) 5.2, msproteomicstools 0.6.0, SWATH2stats 3.12, DIA-Umpire 2.1.3, and ThermoRawFileParser 1.3.4.

The source code and step-by-step instructions to use *glaDIAtor* are provided at https://github.com/elolab/glaDIAtor.

### Laboratory assembled microbial mixture and human fecal samples

The 12mix data was a mixture of twelve different bacterial strains isolated from fecal samples of three human donors grown on fastidious anaerobe agar (LAB 090; LAB M, UK) and annotated by sequencing their 16S-rDNA: *Bacteroides vulgatus*, *Parabacteroides distasonis*, *Enterorhabdus sp*., *Bifidobacterium pseudocatenulatum*, *Escherichia coli*, *Streptococcus agalactiae*, *Bacteroides fragilis*, *Alistipes onderdonkii*, *Collinsella aerofaciens*, *Clostridium sordellii*, *Eubacterium tenue*, and *Bifidobacterium bifidum*. Prior to mixing, the bacterial cell counts were equalized to 10 × 10^8^ cells/ml using flow cytometry and 1 × 10^8^ cells of each isolate were added to the final mixture. Three isolations and mixtures were made, and the mixtures were analyzed in DDA and DIA mode.

The human fecal data contained six human fecal samples from anonymous individuals representing a complex metaproteomic scenario. Each fecal sample was analyzed in DIA mode with a single injection. Additionally, all six samples were pooled together and analyzed in DDA mode with six injections to increase the peptide coverage in the spectral library.

The sample preparation and mass spectrometry analysis were conducted as previously described [10]. Briefly, the protein isolation for the 12mix samples was performed using a Barocycler instrument NEP3229 (Pressure BioSciences) and for the human fecal samples using the NoviPure Microbial Protein Kit (Qiagen) following the manufacturer’s instructions. For each sample, 50 μg of protein was used for trypsin digestion. The proteins were reduced with dithiothreitol and alkylated with iodoacetamide. Trypsin digestion was performed in two steps; first trypsin added in a 1:50 ratio digested for 4 h, and then in a 1:30 ratio overnight at 37 °C. After digestion, the peptides were desalted using a SepPak C18 96-well plate (Waters).

The samples were analyzed using liquid chromatography tandem mass spectrometry (LC–MS/MS) on a nanoflow high-performance liquid chromatography (HPLC) system (EASY-nLC1200) coupled with a Q Exactive HF mass spectrometer (both from Thermo Fisher) and a nano-electrospray ionization source. The digested protein sample (500 ng) was loaded on a trapping column and separated inline on a 15 cm C18 column (Dr. Maisch HPLC GmbH). A mobile phase of water with 0.1% formic acid (solvent A) or acetonitrile/water (80:20 volume/volume) with 0.1% formic acid (solvent B) was applied. Peptides were eluted with a 90 min two-step gradient from 7 to 35% B, followed by wash with 100% B. The mass spectrometry data was acquired automatically using the Thermo Xcalibur 3.1 software (Thermo Fisher). The DDA analysis consisted of an Orbitrap MS survey scan of the mass range 375–1500 m/z with 120 K resolution, AGC target of 3 × 10^6^, and maximum injection time of 50 ms, followed by higher-energy collisional dissociation fragmentation of the 15 most intense peptide ions. The DIA analysis covered the mass range of 400–1000 *m*/*z* through 40 consecutive 15 *m*/*z* isolation windows. The acquisition was performed with 30 K resolution, AGC target of 5 × 10^5^, and automatic maximum injection time.

The mass spectrometry data are available from the ProteomeXchange Consortium via the PRIDE partner repository with the dataset identifier PXD008738.

## Supplementary information

Supplementary Material

## Data Availability

The mass spectrometry data are available from the ProteomeXchange Consortium via the PRIDE partner repository with the dataset identifier PXD008738.
