# Supplementary material for: Introducing untargeted data-independent acquisition for metaproteomics of complex microbial samples
Source: ISME Commun. 2022 Jun 29;2:51. doi: 10.1038/s43705-022-00137-0 (PMC9723653; doi:10.1038/s43705-022-00137-0)
Supplement: Supplementary file 1 — Supplementary Material [file 43705_2022_137_MOESM1_ESM.pdf]

Introducing untargeted data-independent acquisition for metaproteomics of complex microbial samples

Supplementary Material

Sami Pietilä[1,#], Tomi Suomi[1,#], Laura L. Elo[1,2,*]

[1] Turku Bioscience Centre, University of Turku and Åbo Akademi University, FI-20520 Turku, Finland
[2] Institute of Biomedicine, University of Turku, FI-20520 Turku, Finland
[#] Shared first author
* Corresponding author: laura.elo@utu.fi

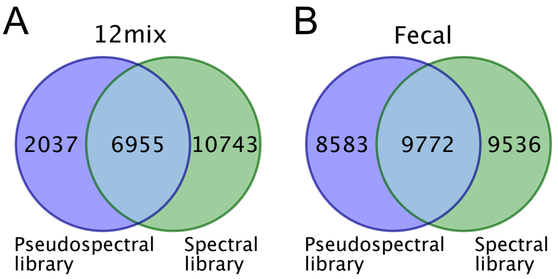

Supplementary Figure 1. Overlap of peptides between the spectral and pseudospectral libraries in the 12mix and the human fecal datasets.

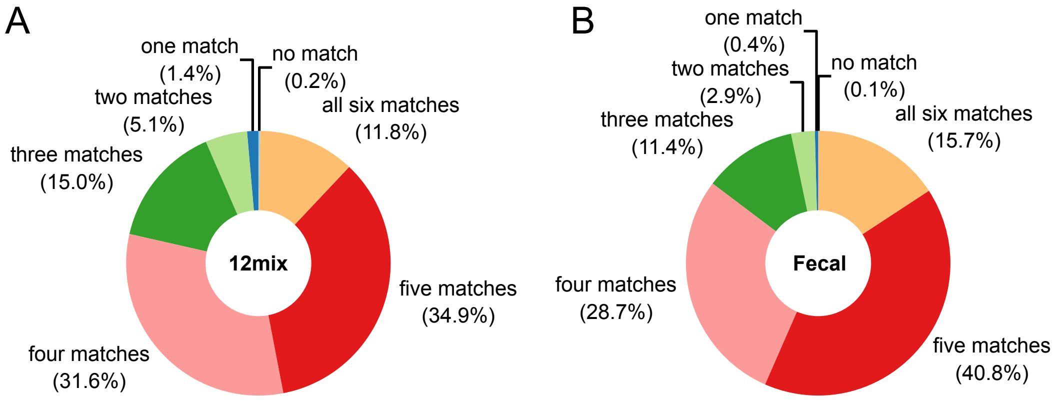

Supplementary Figure 2. The proportion of matching fragment ions in the common peptides between the spectral and pseudospectral libraries in the (**A**) 12mix and (**B**) human fecal datasets. The libraries were built using six fragment ions to represent a peptide.

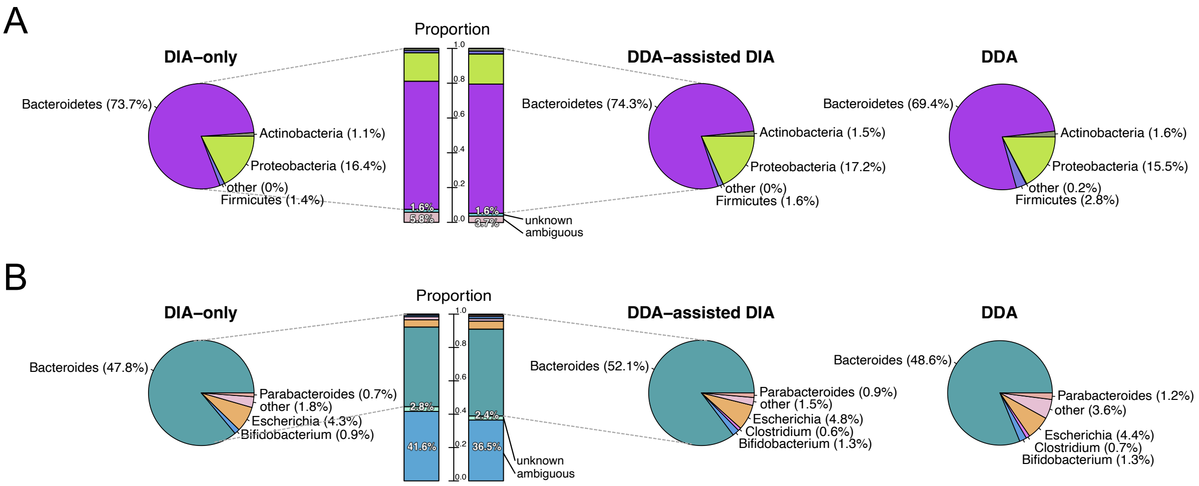

Supplementary Figure 3. Taxonomic profiles of the 12mix samples. **(A)** Phylum-level and **(B)** genus-level taxonomic annotations of the peptides using the DIA-only approach (left panel), the DDA-assisted DIA approach (middle panel), or only the pooled DDA data (right panel). Phyla or genera having less than 0.5% of the total peptides were aggregated to category *other*.

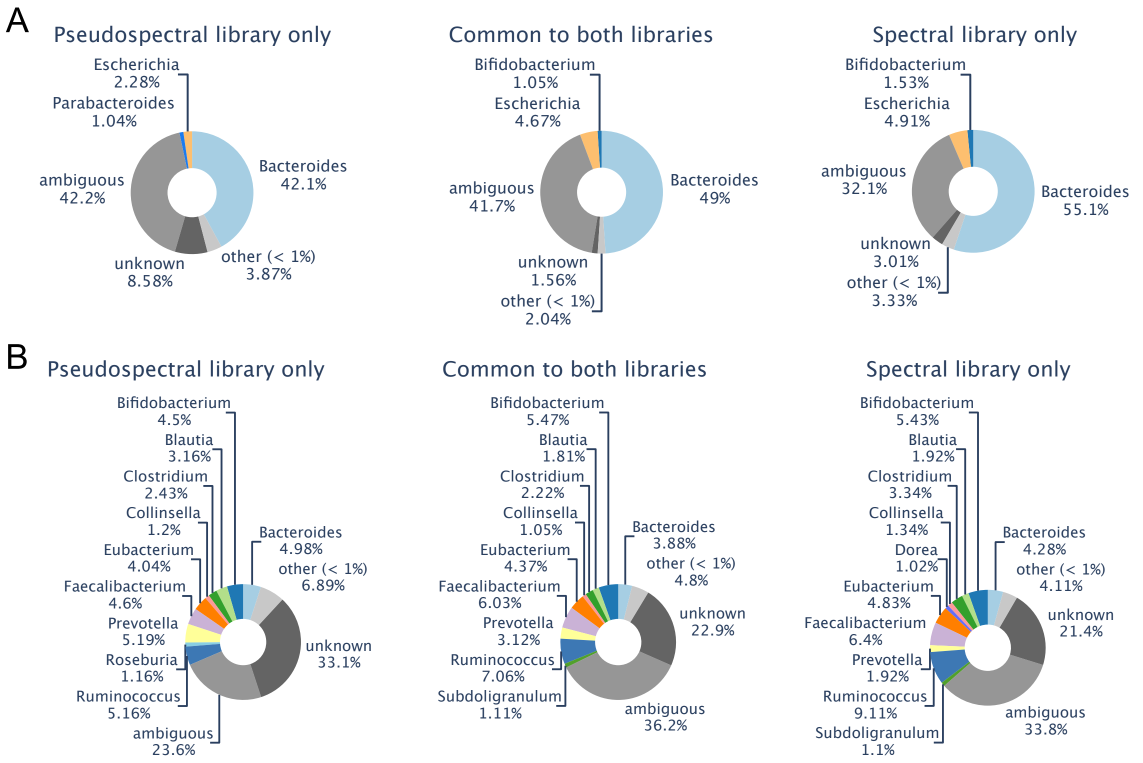

Supplementary Figure 4. Genus-level taxonomic profiles of the peptides in the overlapping and non-overlapping parts of the spectral and pseudospectral libraries in the **(A)** 12mix and **(B)** human fecal datasets. Genera having less than 1% of the total peptides were aggregated to category *other*.

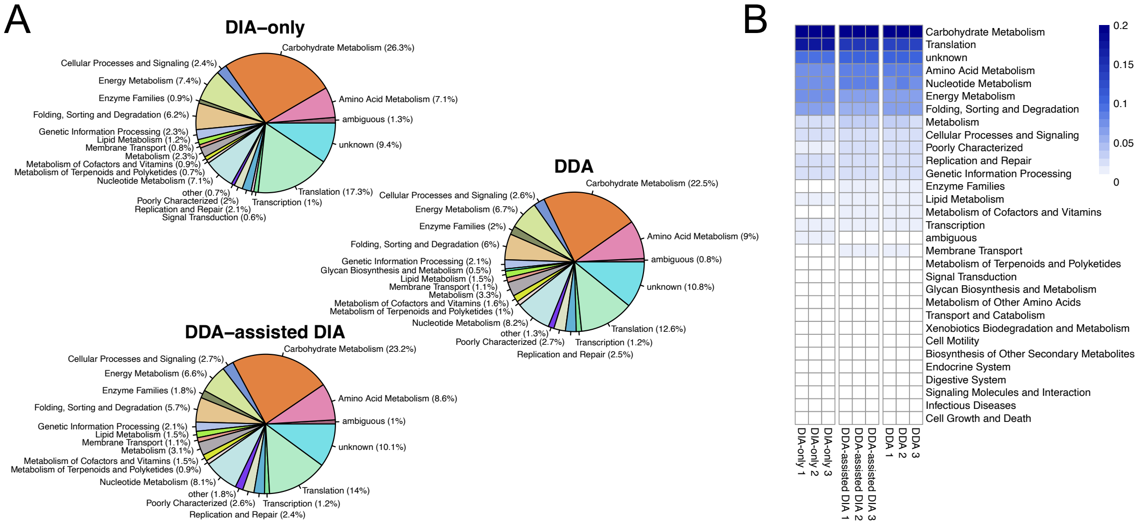

Supplementary Figure 5. Functional profiles of the 12mix samples. **(A)** KEGG functional categories of the peptides using the DIA-only approach the DDA-assisted DIA approach or only the pooled DDA data. Functional categories having less than 0.5% of the total peptides were aggregated to category *other*. **(B)** Heatmaps of the functional profiles of the three replicate samples using the DIA-only and the DDA-assisted DIA approach, as well as the three technical repeats of the pooled sample analyzed with DDA.

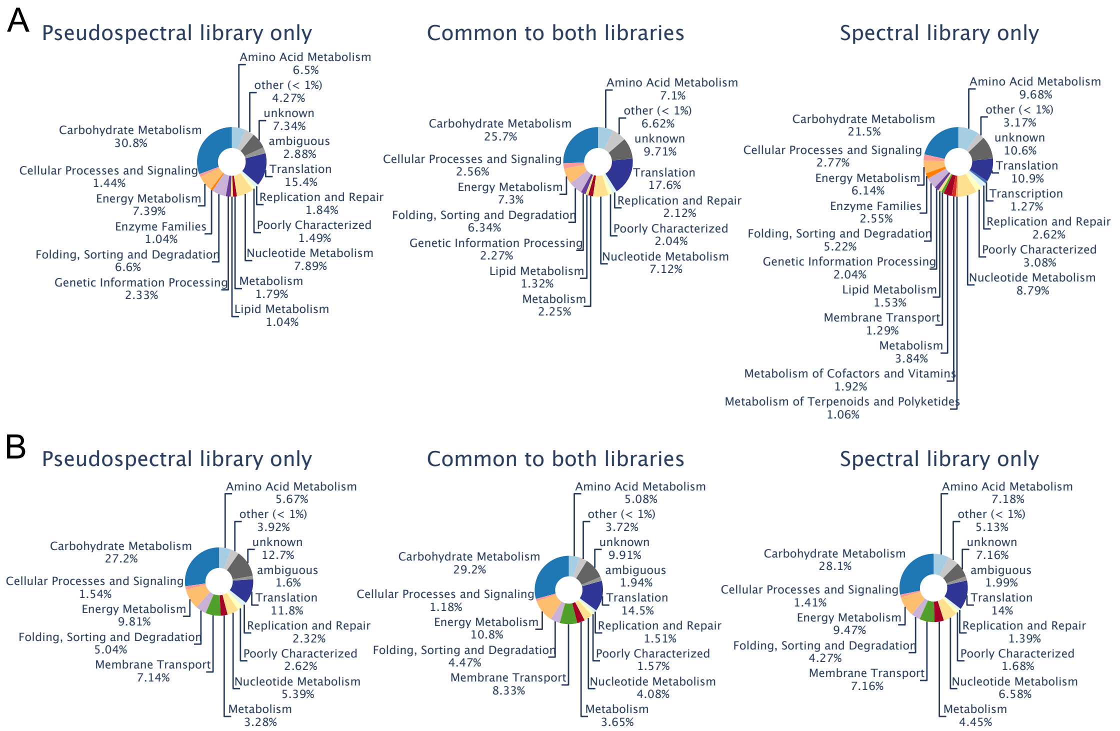

Supplementary Figure 6. Functional profiles of the peptides in the overlapping and non-overlapping parts of the spectral and pseudospectral libraries in the **(A)** 12mix and **(B)** human fecal datasets using KEGG functional categories. Genera having less than 1% of the total peptides were aggregated to category *other*.

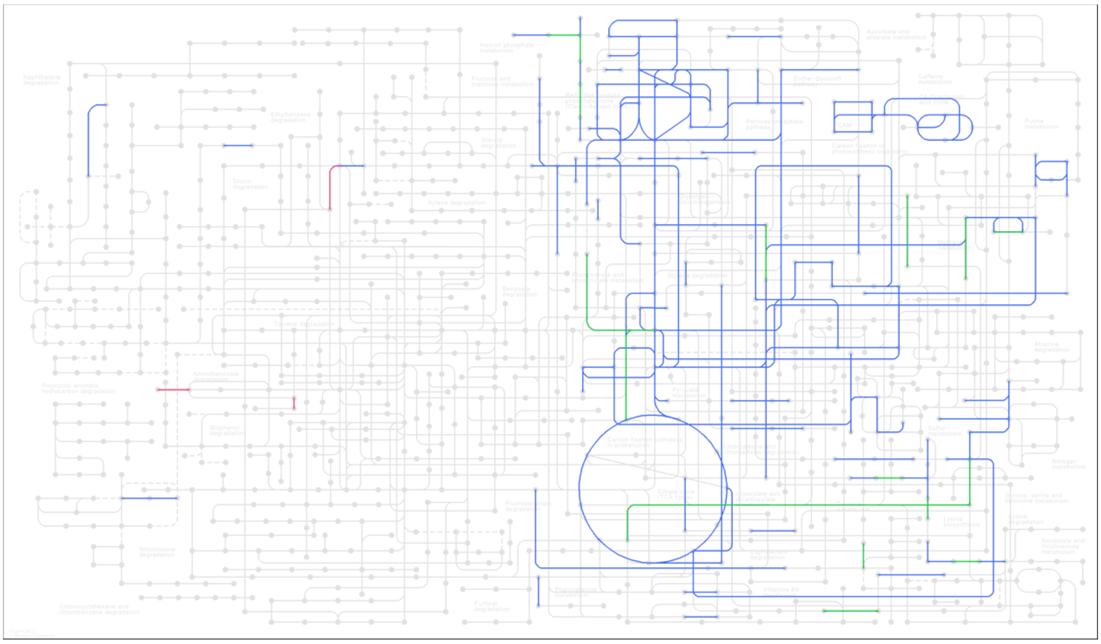

Supplementary Figure 7. Mapping of the peptides identified in the human fecal samples to the KEGG map of *Microbial metabolism in diverse environments*. Green color corresponds to peptides that were only identified using the DIA-only approach, red color those that were only identified using the DDA-assisted DIA approach, and blue color those that were identified using both approaches.

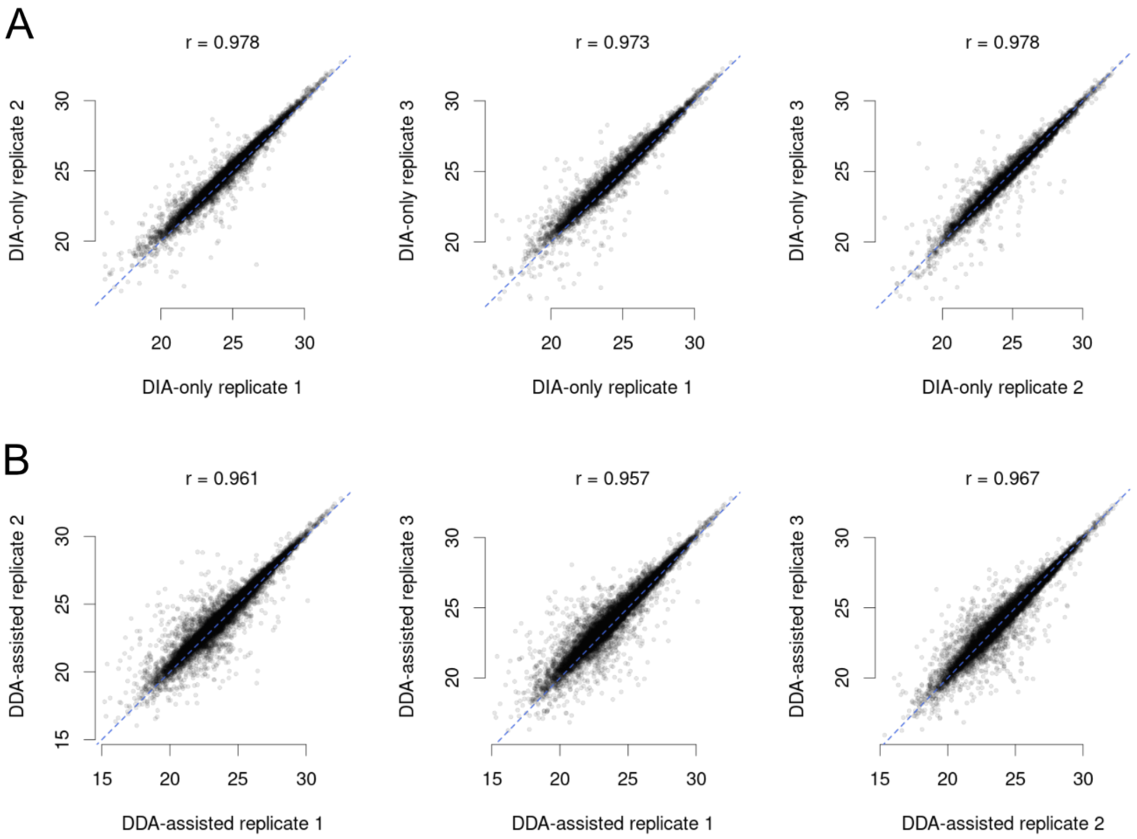

Supplementary Figure 8. Correlations of peptide quantifications between each pairwise comparison of technical replicates of the 12mix samples using (**A**) the DIA-only or (**B**) the DDA-assisted approach. All Pearson correlation coefficients (r) were highly significant ($p < 0.001$).

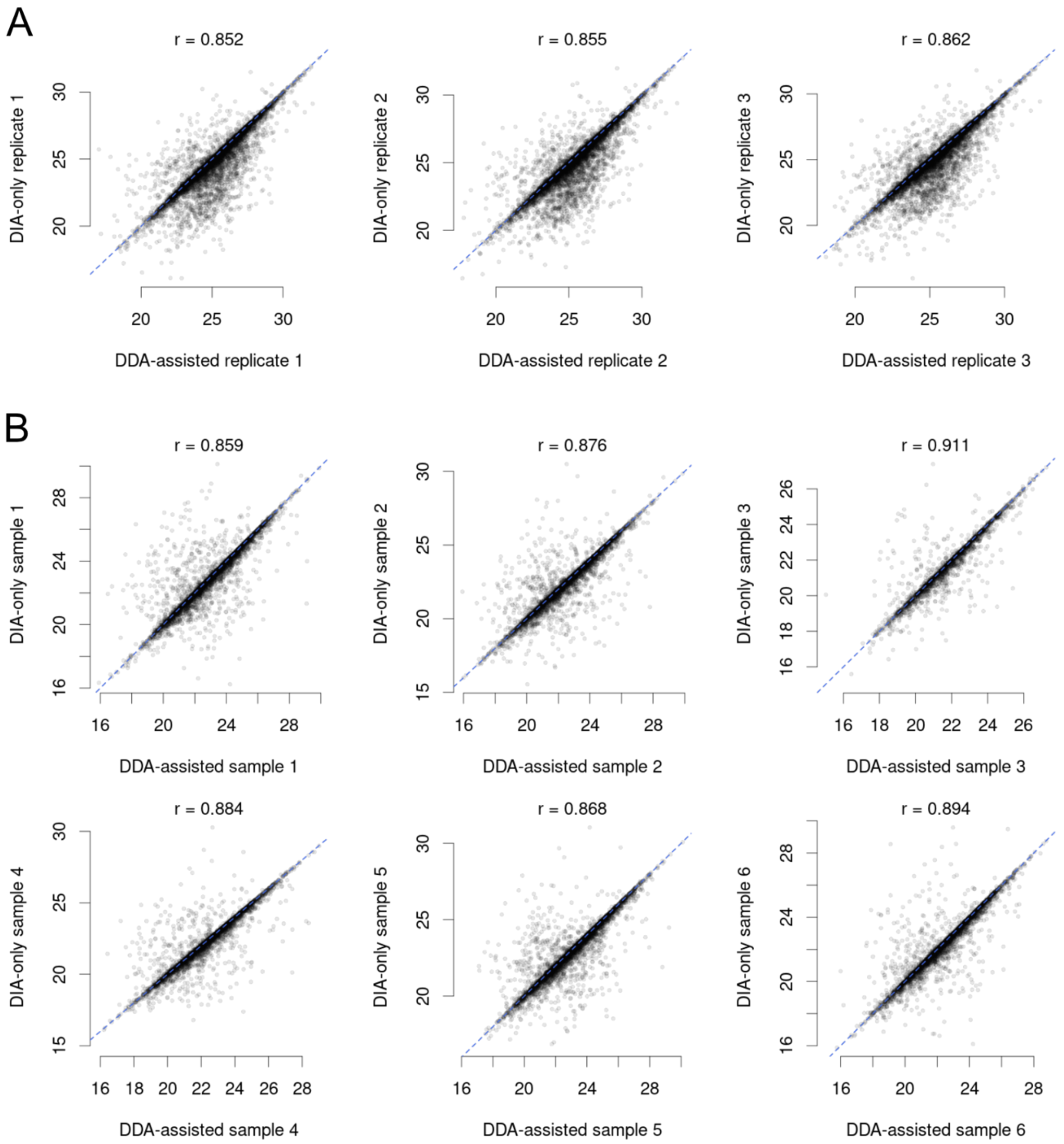

Supplementary Figure 9. Correlations of peptide quantifications between the DIA-only and the DDA-assisted approach in (**A**) 12mix and (**B**) human fecal samples. All Pearson correlation coefficients (r) were highly significant ($p < 0.001$).

Supplementary Table 1. The 12mix dataset files. The files have been deposited to the ProteomeXchange Consortium via the PRIDE partner repository with the dataset identifier, PXD008738.

File Type	File Name	Description
DDA	170412_12mix_DDA_library_stock_2_2.raw	Pooled spectra for library
DDA	170412_12mix_DDA_library_stock_2_3.raw	Pooled spectra for library
DDA	170412_12mix_DDA_library_stock_2_4.raw	Pooled spectra for library
DIA	170413_12mix_DIA_14.raw	Replicated Sample #1
DIA	170413_12mix_DIA_15.raw	Replicated Sample #2
DIA	170413_12mix_DIA_16.raw	Replicated Sample #3

Supplementary Table 2. The human fecal dataset files. The files have been deposited to the ProteomeXchange Consortium via the PRIDE partner repository with the dataset identifier PXD008738.

File Type	File Name	Description
DDA	170825_HF_1_6_pool_2ug_DDA_library_1column_1.raw	Pooled spectra for library
DDA	170825_HF_1_6_pool_2ug_DDA_library_1column_2.raw	Pooled spectra for library
DDA	170825_HF_1_6_pool_2ug_DDA_library_1column_3.raw	Pooled spectra for library
DDA	170825_HF_1_6_pool_2ug_DDA_library_1column_4.raw	Pooled spectra for library
DDA	170825_HF_1_6_pool_2ug_DDA_library_1column_5.raw	Pooled spectra for library
DDA	170825_HF_1_6_pool_2ug_DDA_library_1column_6.raw	Pooled spectra for library
DIA	170825_HF_1ug_DIA_1column_1.raw	Sample 1
DIA	170825_HF_1ug_DIA_1column_2.raw	Sample 2
DIA	170825_HF_1ug_DIA_1column_3.raw	Sample 3
DIA	170825_HF_1ug_DIA_1column_4.raw	Sample 4
DIA	170825_HF_1ug_DIA_1column_5.raw	Sample 5
DIA	170825_HF_1ug_DIA_1column_6.raw	Sample 6
